# Quality of Life and Its Association with Physical Activity among Different Types of Cancer Survivors

**DOI:** 10.1371/journal.pone.0164971

**Published:** 2016-11-03

**Authors:** Furong Tang, Jiwei Wang, Zheng Tang, Mei Kang, Qinglong Deng, Jinming Yu

**Affiliations:** Institute of Epidemiology and Health Statistics, School of Public Health, Fudan University, Shanghai, China; University of Rochester, UNITED STATES

## Abstract

**Purpose:**

The main goal of this study was to compare the quality of life (QOL) and its association with physical activity (PA) among patients diagnosed with different types of cancer. Based on the results, we tentatively present suggestions for the cancer health care model.

**Method:**

A cross-sectional study was conducted with 2915 cancer survivors recruited from multi-community cancer rehabilitation centers, all of which were affiliated with the Shanghai Cancer Rehabilitation Club. We collected data including socio-demographic characteristics and information about PA. All the subjects included were asked to complete the European Organization for Research and Treatment Quality of Life Questionnaires (EORTC QLQ-C30) and Functional Assessment of Cancer Therapy—General Questionnaire (FACT-G). Multiple linear regression models were employed to control the potential confounding factors.

**Results:**

Lung cancer survivors reported the worst dyspnea. Colorectal cancer survivors claimed the highest level of constipation and diarrhea. Liver cancer survivors indicated greatest loss of appetite and financial difficulties. Generally, survivors with PA tended to reported better QOL, although these associations among liver cancer survivors were not statistically significant. Moreover, survivors of all cancer types who performed PA did not report significant lower level of constipation or diarrhea. The relationship between PA frequency and QOL among cancer survivors remained unexplored.

**Conclusions:**

Both QOL and its association with PA vary among survivors of different cancer types. The detailed results can assist clinicians and public health practitioners with improving health care management.

## Introduction

Cancer has become a major cause of death all over the world. Despite the high mortality of cancer, patients diagnosed with cancer survive much longer than ever before due to early detection [1–3], effective treatment [4, 5] and improved health management [6]. However, compared with the general population, most cancer survivors experience poorer quality of life (QOL) in both physical and psychological aspects after cancer diagnosis and its treatment [7, 8]. Meanwhile, longer survival time makes QOL an even more important outcome measure for the population with metastatic disease, for whom a cure is probably not the goal. The significance of QOL of cancer survivors has been increasing recognized, as reflected by the growing number of investigations that have included QOL measures as part of their endpoints [9–13].

Recently, more investigators have realized the importance of health management for cancer survivors and have put forward strategies that aim to improve QOL among those patients [14–16]. Physical activity (PA) has been increasingly regarded as a non-pharmacologic intervention for cancer patients to combat both the physiologic and psychologic effects of treatment [17]. In addition, evidence has shown that PA may improve multiple QOL measures among cancer survivors [18]. In a previously published article related to the present study, survivors with PA reported better QOL on many aspects among lung cancer survivors [19]. However, according to the results of some other studies, the implication of PA on QOL among cancer survivors remains unclear [20, 21]. The differences of these conclusions imply that the relationship between PA and QOL may differ among patients diagnosed with different types of cancers and thus inspire us to explore the relationship. Therefore, to better explore the discrepancy, we included lung, cervical, ovarian, endometrial, colorectal and liver cancer survivors in this study. The main goal of our study was to investigate both QOL and its relationship with PA among patients with different types of cancer.

In China, the health-related management of cancer usually focuses on recurrence and metastasis. Far less attention has been paid to QOL among cancer survivors, let alone the diversity of QOL and its association with PA among patients diagnosed with different types of cancer. Therefore, the results of this study will have both clinical and public health implications, taking a further step forward in this research area.

## Methods

### Recruitment

Cancer survivors were consecutively recruited from April to July 2013 from multi-community cancer rehabilitation centers, all of which were affiliated with the Shanghai Cancer Rehabilitation Club, Shanghai, China. The inclusion criteria were as follows: (1) a clinical diagnosis of lung, cervical, ovarian, endometrial, colorectal or liver cancer; (2) the ability to speak, read and write Chinese; (3) the ability and willingness to provide written informed consent; (4) no substantially impaired cognitive functions caused by major disabling psychiatric or medical conditions.

Ultimately, 3392 cancer survivors participated in this study and formed our final sample, including 701 lung cancer survivors, 224 cervical cancer survivors, 299 ovarian cancer survivors, 75 endometrial cancer survivors, 1398 colorectal cancer survivors and 218 liver cancer survivors. Cervical cancer, ovarian cancer and endometrial cancer were collectively called gynecological cancer in this study, and gynecological cancer was analyzed as a single entity. The questionnaires were completed under instruction. Ethical approval to conduct this study was granted by the Medical Research Ethics Committee of the School of Public Health, Fudan University (protocol number RB #201304–0450). Written informed consent was obtained from each participant in advance.

### Instruments

#### Socio-demographic characteristics and health-related conditions

We collected demographic information about gender, age, socioeconomic status, educational level, body mass index (BMI), and other socio-demographic information. Participants were asked to choose “yes” or “no” to indicate whether they suffered from comorbid chronic diseases (CCDs), including respiratory diseases, heart diseases, hypertension, digestive diseases, diabetes mellitus, and musculoskeletal diseases.

#### Physical activity

Subjects were asked the question, “Did you engage in moderate-intensity physical activity (such as badminton, vigorous walking, table tennis, running, or tai chi) for a minimum of 30 minutes once a week during the last month?” and, “If you did, how many times did you engage in these activities every week?”

#### QOL measurements

The EORTC QLQ-C30 (version 3.0) was designed and validated to assess the QOL of cancer survivors [22]. The EORTC QLQ-C30 is a 30-item self-reporting tool composed of five functional scales (cognitive, physical, emotional, role, and social), nine symptom scales (pain, insomnia, fatigue, nausea and vomiting, appetite loss, dyspnea, constipation, financial difficulties, and diarrhea), and one global health status or QOL item. According to the guidelines provided by the EORTC, scores range from 0 to 100 after linear transformation and they are continuous. A higher score on the functional scales corresponds to a healthier level of functioning, and a higher score on the global health status indicates a better QOL. A high score on the symptom scale indicates a higher level of problems or symptoms.

The FACT-G is a 27-item self-reporting instrument. All of the item scales range from 0 to 4 and can be divided into four domains: physical, emotional, social, and functional well-being. The FACT-G total scores range from 0 to 108 and they are continuous, with higher scores indicating better QOL.

The full version of EORTC QLQ-C30 (version 3.0) and FACT-G can be found in S1 and S2 Files respectively.

### Statistical analysis

Student’s t-tests and ANOVA tests were utilized for continuous variables. A statistical significance level of alpha = 0.05 is used to decide about the significance of p-values.

PA was defined as a dichotomous variable (yes or no) in statistical analyses. The frequency of PA was also defined as a dichotomous variable and divided into two categories: “1–4 times/week” and “≥5 times/week”. Multivariate linear regression models were used to compute regression coefficients (β) as estimates of the mean difference of the QOL scores, after adjusting for potential confounding variables. The following potentially confounding variables were included in the regression models: age, gender, BMI, years after diagnosis, household income, education level, marital status, treatment, CCDs, the number of CCDs, PA and PA frequency. BMI was categorized according to a report of WHO [23]. When evaluating the relationship between PA or PA frequency and QOL, PA and PA frequency were regarded as independent variables instead of confounding variables.

All statistical analyses were performed using SPSS 19.0.

## Results

### Characteristics of subjects

The socio-demographic and health-related characteristics and global QOL for the cancer survivors are shown in Table 1. Cancer survivors who were married and those with a BMI from 18.5 to 25 were prevalent in this sample. Lung cancer survivors who were married reported higher QOL than their counterparts who were not married. In the subgroups of treatment/ CCD, global QOL was described as the form of “The differences in mean global QOL score between cancer survivors with and without treatment/CCD (Mean score of global QOL measure among cancer survivors without treatment/CCD)”. The mean scores were continuous and student’s t-tests were applied to test the mean scores. Surgery was reported to improve QOL for lung and liver cancer survivors. CCDs were observed to have more negative influence on QOL among gynecological cancer survivors than on survivors of other cancers. As the number of CCDs increased, QOL tended to decrease among cancer survivors of all types.

**Table 1:** Socio-demographic and health-related information for cancer survivors and the global QOL item for subgroups.

	lung cancer survivors		gynecological cancer survivors		colorectal cancer survivors		liver cancer survivors	
	N(%)	global QOL^a^	N(%)	global QOL^a^	N(%)	global QOL^a^	N(%)	global QOL^a^
Gender								
Male	382(54.5)	61.7±26.2	0	NA	670(47.9)	65.3±24.9	148(67.9)	59.1±24.3
Female	319(45.5)	60.0±21.0	598(100)	61.3±23.3	728(52.1)	59.1±25.0	70(32.1)	60.1±22.4
		P = 0.379		NA		P<0.000		P = 0.805
Age								
<60	229(32.7)	63.5±23.8	339(56.7)	61.9±23.1	388(27.8)	63.1±24.7	77(35.3)	56.1±24.1
≥60	472(67.3)	59.7±24.0	259(43.3)	60.5±23.5	1010(72.2)	61.6±25.2	141(64.7)	61.3±23.3
		P = 0.084		P = 0.510		P = 0.359		P = 0.176
BMI								
<18.5	29(4.1)	56.3±18.4	29(4.8)	55.9±22.0	59(4.2)	53.1±27.1	9(4.1)	38.1±26.7
18.5≤BMI<25	490(70.0)	60.4±23.7	360(60.2)	61.6±23.1	946(67.7)	61.8±24.7	169(77.5)	59.4±23.1
25≤BMI<30	167(23.8)	62.3±26.1	185(31.0)	61.6±24.1	350(25.0)	64.9±25.0	38(17.4)	63.1±23.1
≥30	15(2.1)	69.9±18.5	24(4.0)	62.9±23.0	43(3.1)	55.5±27.9	2(0.9)	83.3±23.6
		P = 0.340		P = 0.653		P = 0.006		P = 0.036
Education								
Less than high school	331(47.2)	62.4±25.1	304(50.8)	61.8±25.0	681(48.7)	62.5±27.0	93(42.7)	62.4±24.4
Junior school	312(44.5)	60.1±23.3	268(44.8)	61.3±21.3	627(44.8)	61.7±24.0	112(51.4)	56.3±22.3
More than junior school	58(8.3)	58.2±22.0	26(4.3)	57.3±23.0	90(6.4)	62.0±18.8	13(6.0)	67.6±28.4
		P = 0.382		P = 0.669		P = 0.804		P = 0.153
Marital Status								
Married	642(91.6)	61.6±23.7	505(84.4)	61.4±23.8	1239(88.6)	61.8±24.9	190(87.2)	60.7±23.4
Widowed/divorced/single	59(8.4)	53.1±26.7	94(15.6)	60.9±20.3	159(11.4)	63.8±26.7	28(12.8)	50.8±24.0
		P = 0.023		P = 0.872		P = 0.389		P = 0.065
Income (yen/month)								
<2000	173(24.7)	59.1±25.7	187(31.3)	61.8±25.5	348(24.9)	63.9±27.3	46(21.1)	59.2±24.4
2000–4000	399(56.9)	62.0±24.1	322(53.8)	60.4±21.9	805(57.6)	61.3±23.5	135(61.9)	57.5±21.6
>4000	129(18.4)	60.2±21.3	89(14.9)	63.7±23.6	245(17.5)	61.5±26.7	37(17.0)	65.8±23.6
		P = 0.457		P = 0.533		P = 0.343		P = 0.239
TAD (year)								
≤2	170(24.3)	60.5±24.6	127(21.2)	64.6±23.8	283(20.2)	60.4±27.2	36(16.5)	57.4±27.0
3–5	225(32.1)	62.1±22.4	180(30.1)	61.2±24.2	422(30.2)	64.2±24.7	59(27.1)	56.3±24.9
6–9	175(25.0)	62.4±24.1	115(19.2)	63.8±23.9	305(21.8)	60.3±24.4	57(26.1)	64.2±21.5
>10	131(18.7)	57.9±24.5	162(27.1)	57.6±21.7	329(23.5)	62.8±24.0	59(27.1)	59.1±21.8
		P = 0.389		P = 0.104		P = 0.172		P = 0.433
Treatment								
Surgery	566(80.7)	8.5(53.9)**	532(89.0)	-3.2(64.2)	1288(92.1)	-2.3(64.2)	173(79.4)	10.6(51.0)*
Radiotherapy	194(27.7)	-1.8(24.4)	179(29.9)	-0.3(23.4)	251(18.0)	4.7(24.0)***	39(17.9)	1.6(22.8)**
Chemotherapy	522(74.5)	-1.9(62.4)	435(72.7)	3.4(58.8)	1133(81.0)	-0.6(62.5)	94(43.1)	-6.7(62.4)
Other treatments	409(58.3)	1.4(60.0)	293(49.0)	-6.2(64.4)**	803(57.4)	-1.9(63.1)	122(56.0)	1.7(58.5)
CCD								
Hypertension	261(37.2)	-2.1(61.7)	184(30.8)	-4.9(62.8)*	538(38.5)	-3.5(63.4)*	76(34.9)	-5.9(61.5)
Diabetes	102(14.6)	-7.7(62.1)**	106(17.7)	-6.8(62.6)*	250(17.9)	-2.9(62.6)	30(13.8)	-0.8(59.5)
Heart and Cardiovascular	166(23.7)	-8.0(62.8)**	147(24.6)	-7.9(63.0)**	304(21.7)	-8.7(63.9)***	39(17.9)	-13.8(61.7)**
Respiratory disease	277(39.5)	-6.4(63.5)**	49(8.2)	-14.0(62.4)***	169(12.1)	-15.6(63.9)***	35(16.1)	-5.3(60.3)
Digestive diseases	319(45.5)	-2.3(62.0)	297(49.7)	-4.54(63.8)*	723(51.7)	-6.8(65.7)***	134(61.5)	-9.0(65.0)*
Musculoskeletal diseases	185(26.4)	-9.0(63.4)***	191(31.9)	-11.2(64.9)***	409(29.3)	-9.4(64.9)***	46(21.1)	-9.3(61.7)*
No. of CCDs								
0	137(19.5)	67.9±23.8	138(23.1)	65.8±24.7	315(22.5)	68.8±24.0	43(19.7)	64.8±27.2
1	125(17.8)	65.2±24.2	138(23.1)	65.5±22.1	375(26.8)	65.2±23.9	64(29.4)	63.4±23.0
2	150(21.4)	57.0±25.3	119(19.9)	61.2±21.8	324(23.2)	60.7±24.9	62(28.4)	58.3±20.1
≥3	348(41.2)	56.7±21.5	203(33.9)	52.2±22.0	384(24.5)	55.0±25.3	49(22.5)	51.1±23.8
		P_anova_<0.000		P_anova_<0.000		P_anova_<0.000		P_anova_<0.000

Abbreviations: BMI, body mass index; TAD, time after diagnosis; CCD, comorbid chronic disease.

^a^ Global QOL was described in the form “mean ± standard deviation” for the subgroups of gender, age, BMI, education, marital status, TAD and number of CCDs. In the subgroups of treatment/CCD, global QOL was described as “The differences in mean global QOL score between cancer survivors with and without treatment/CCD (Mean score of global QOL measure among cancer survivors without treatment/CCD)”. The mean scores were continuous and student’s t-tests were applied to test the mean scores,

*p<0.05,

**p<0.01,

***p<0.001.

### QOL among cancer survivors

Adjusted QOL measures are shown in Table 2. Lung cancer survivors reported worse physical functioning than gynecological or colorectal cancer survivors. Lung cancer survivors reported higher scores for dyspnea than other survivors. Liver cancer survivors reported the highest level of appetite loss, followed by colorectal cancer survivors. Colorectal cancer survivors were observed to have the most serious diarrhea, while breast lung survivors reported the least. Liver cancer survivors indicated the severest level of financial difficulties. No statistically significant differences between any two types of cancer survivors were observed in cognitive functioning, pain or insomnia. According to the results of FACT-G, liver cancer survivors performed the worst in emotional well-being compared with all other types of cancer survivors.

**Table 2:** Adjusted quality of life for cancer survivors.

	lung cancer survivors	gynecological cancer survivors	colorectal cancer survivors	liver cancer survivors
EORTC QLQ-C30				
PF	79.2±0.7^ab^	81.3±0.6^a^	81.0±0.5^b^	81.5±1.1
RF	87.0±0.8^a^	89.3±0.8^a^	88.3±0.6	86.3±1.4
EF	84.2±0.7	83.5±0.7^e^	83.2±0.5	81.6±1.2
CF	78.0±0.7	77.8±0.8	77.9±0.6	78.0±1.3
SF	74.6±1.0	76.4±1.0^e^	76.7±0.7^f^	72.5±1.7^ef^
QL	60.9±1.1	61.4±1.1^e^	61.0±0.8^f^	56.8±1.9^ef^
FA	31.3±0.8	30.0±0.8	29.6±0.6	31.0±1.4
NV	4.0±0.5	3.7±0.5	4.3±0.3	5.0±0.8
PA	18.1±0.8	17.7±0.8	17.6±0.6	18.8±1.4
DY	22.0±0.8^abc^	15.8±0.4^a^	16.2±0.6^b^	17.8±1.5^c^
SL	21.4±1.0	21.2±1.0	20.8±0.7	20.1±1.8
AP	11.1±0.8^c^	9.1±0.7^de^	11.6±0.6^df^	15.4±1.3^cef^
CO	10.2±0.9^b^	11.9±0.8^d^	15.9±0.6^bdf^	10.7±1.5^f^
DI	7.6±0.7^bc^	8.2±0.7^d^	12.6±0.5^bd^	10.8±1.2^c^
FI	36.5±1.3^b^	33.2±1.3^e^	33.0±0.9^bf^	38.7±2.2^ef^
FACT-G				
PWB	22.8±0.2	22.8±0.2	22.8±0.1	22.2±0.4
SWB	18.5±0.3	17.7±0.3^d^	18.5±0.2^d^	18.1±0.5
EWB	18.8±0.2^c^	18.8±0.2^e^	18.7±0.1^f^	18.0±0.3^cef^
FWB	15.0±0.3	15.2±0.3	15.0±0.2	14.9±0.5
FACT-G	75.1±0.7	74.5±0.7	74.8±0.5	73.2±1.3

Multiple linear regression adjusted for the potential influence of gender, age, BMI, education level, marital status, household income, time after diagnosis, treatment, comorbid chronic diseases, the number of comorbid chronic diseases, physical activity and physical activity frequency.

Abbreviations: EORTC QLQ-C30, the European Organization for Research and Treatment Quality of Life Questionnaires; PF, Physical Functioning; RF, Role Functioning; EF, Emotional Functioning; CF, Cognitive Functioning; SF, Social Functioning; QL, Global Health; FA, Fatigue; NV, Nausea and Vomiting; PA, Pain; DY, Dyspnea; SL, Insomnia; AP, Appetite Loss; CO, Constipation; DI, Diarrhea; FI, Financial Difficulties; FACT-G, Functional Assessment of Cancer Therapy—General Questionnaire; PWB, Physical Well-being; SWB, Social/family Well-being; EWB, Emotional Well-being; FWB, Functional Well-being.

Student’s t-tests were applied to test the mean scores between survivors of different cancer types. Superscripts were used to indicate statistically significant differences: a: lung vs gynecological; b: lung vs colorectal; c: lung vs liver; d: gynecological vs colorectal; e: gynecological vs liver; f: colorectal vs liver.

### The relationship between PA and QOL

QOL measures for cancer survivors that performed and did not perform PA are shown in Table 3. When focusing on the functional scales in EORTC QLQ-C30, subjects with PA reported higher scores for all the functional scales and the global health status than their counterparts without PA, implying better QOL for these items. However, many of these associations among liver cancer survivors showed no statistically significant differences. Despite these findings, survivors of all cancer types who performed PA reported statistically significant higher scores in physical functioning than counterparts.

**Table 3:** Quality of life for cancer survivors with and without physical activity.

	lung cancer survivors	gynecological cancer survivors	colorectal cancer survivors	liver cancer survivors
	PA, n = 497^a^(no PA, n = 204)^b^	PA, n = 407^a^ (no PA, n = 191)^b^	PA, n = 1010^a^ (no PA, n = 388)^b^	PA, n = 145^a^ (no PA, n = 73)^b^
EORTC QLQ-C30				
PF	6.0(75.0)***	7.0(76.4)***	8.0(76.4)***	7.5(78.6)***
RF	8.7(80.4)***	8.1(83.847)***	7.1(84.1)***	5.7(84.4)*
EF	3.7(81.6)*	4.8(79.7)**	4.8(81.4)***	4.2(80.5)
CF	6.0(73.5)***	3.9(74.7)*	4.8(75.5)***	3.9(76.5)
SF	7.1(68.5)**	6.0(72.1)**	5.3(74.3)***	3.0(71.5)
QL	9.1(54.3)***	3.9(58.6)	6.0(57.8)***	3.1(57.2)
FA	-7.0(36.0)***	-7.2(34.9)***	-5.6(32.0)***	-4.1(31.6)
NV	-3.1(6.8)**	-1.6(4.9)	-3.5(6.2)***	-3.6(7.1)
PA	-4.3(21.3)**	-6.0(22.1)**	-4.3(19.7)***	-3.5(19.6)
DY	-4.4(27.4)*	-3.9(18.0)*	-4.3(18.4)**	-5.2(20.3)
SL	-5.3(24.7)**	-2.5(23.3)	-3.0(21.1)*	-8.2(23.1)*
AP	-4.4(15.0)**	-3.0(11.4)	-5.6(15.4)***	-4.9(17.5)
CO	-3.0(12.5)	-4.2(14.7)*	0.3(15.0)	-1.9(11.5)
DI	-0.2(8.3)	-1.8(9.5)	-1.7(13.1)	4.8(7.2)
FI	-5.7(40.2)*	-2.174(36.1)	-2.9(32.3)	7.7(30.0)
FACT-G				
PWB	1.4(21.6)**	1.6(21.6)***	1.4(22.1)***	0.4(22.4)
SWB	1.5(17.0)*	1.5(16.7)*	1.8(17.0)***	0.5(17.4)
EWB	0.5(18.3)	0.9(18.0)*	0.9(18.4)***	0.5(17.9)
FWB	2.2(13.0)***	1.0(14.3)	2.2(13.7)***	1.2(14.2)
FACT-G	5.7(70.0)***	4.9(70.7)**	6.2(71.2)***	2.61(71.8)

Multiple linear regression adjusted for the potential influence of gender, age, BMI, education level, marital status, household income, time after diagnosis, treatment, comorbid chronic diseases and the number of comorbid chronic diseases.

Abbreviations: PA, physical activity; EORTC, QLQ-C30 the European Organization for Research and Treatment Quality of Life Questionnaires; PF, Physical Functioning; RF, Role Functioning; EF, Emotional Functioning; CF, Cognitive Functioning; SF, Social Functioning; QL, Global Health; FA, Fatigue; NV, Nausea and Vomiting; PA, Pain; DY Dyspnea; SL, Insomnia; AP, Appetite Loss; CO, Constipation; DI, Diarrhea; FI, Financial Difficulties; FACT-G, Functional Assessment of Cancer Therapy—General Questionnaire; PWB, Physical Well-being; SWB, Social/family Well-being; EWB, Emotional Well-being; FWB, Functional Well-being.

^a^ The differences in mean QOL score between cancer survivors with and without PA.

^b^ Mean score of QOL measure among cancer survivors without PA.

*p<0.05,

**p<0.01,

***p<0.001.

Cancer survivors who performed PA generally reported lower scores on the symptom scales in EORTC QLQ-C30, indicating a lower level of problems or symptoms. However, of the nine symptom scales, only one (insomnia) showed a significantly lower score among liver cancer survivors who performed PA. Generally, cancer survivors of all types who performed PA did not report statistically significant improvements in constipation, diarrhea or financial difficulties.

According to the results of FACT-G, survivors with PA reported significant higher scores on all of the scales for lung, gynecological or colorectal cancer survivors. However, the relationship between PA and QOL was not statistically significant among liver cancer survivors.

### The relationship between PA frequency and QOL among cancer survivors with PA

QOL measures for cancer survivors with higher and lower PA frequencies are shown in Table 4**.** According to the results of the EORTC QLQ-C30, subjects with higher PA frequency generally reported higher scores for the functional scales than their counterparts with a lower PA frequency, implying better QOL for these items. The relationship between PA frequency and QOL remains unexplored on most of the functional scales among gynecological, colorectal or liver cancer survivors, due to the absence of statistically significant differences.

**Table 4:** Quality of life for cancer survivors with different PA frequencies.

	lung cancer survivors	gynecological cancer survivors	colorectal cancer survivors	liver cancer survivors
	≥5 times/week, n = 361^a^ (1–4 times/week, n = 136)^b^	≥5 times/week, n = 211^a^ (1–4 times/week, n = 196)^b^	≥5 times/week, n = 639^a^ (1–4 times/week, n = 371)^b^	≥5 times/week, n = 98^a^ (1–4 times/week, n = 47)^b^
EORTC QLQ-C30				
PF	4.9(78.4)**	0.3(83.1)	1.9(83.1)*	1.4(85.3)
RF	1.5(87.9)	-0.2(92.2)	-0.1(91.1)	5.5(86.5)
EF	3.8(83.2)*	1.5(83.1)	0(86.3)	0.9(84.5)
CF	4.1(76.9)*	1.9(77.1)	0.8(79.8)	2.6(78.7)
SF	2.8(73.2)	-3.2(78.6)	0.5(79.5)	4.3(72.9)
QL	0.7(63.4)	-3.4(64.1)	3.8(61.1)*	-0.4(60.4)
FA	-4.0(32.0)*	2.3(26.7)	-0.9(27.1)	-1.3(27.7)
NV	-1.8(4.3)	0.7(2.7)	-0.6(2.9)	0.22(3.5)
PA	-1.5(17.8)	1.0(16.0)	-0.2(15.8)	0(15.6)
DY	-2.5(25.0)	0.7(13.5)	-1.7(15.2)	-0.5(14.6)
SL	-2.6(21.4)	6.3(17.4)*	0.7(17.4)	2.9(13.4)
AP	-4.6(12.5)**	1.2(7.7)	0.1(8.5)	-1.8(13.7)
CO	-6.0(12.8)**	-1.4(11.4)	1.4(14.6)	2.3(8.3)
DI	-1.2(8.8)	1.4(7.4)	1.5(10.9)	-2.7(13.7)
FI	-1.9(35.5)	5.5(32.2)	0.3(28.6)	-6.0(40.5)
FACT-G				
PWB	1.1(22.3)*	-0.7(23.4)	0.2(23.5)	-1.0(23.5)
SWB	0.9(18.1)	0.1(18.3)	1.2(18.2)*	0(18.1)
EWB	0.6(18.6)	0(18.8)	0.3(19.2)	-0.6(19.1)
FWB	0.9(14.8)	-0.8(15.9)	1.6(14.9)**	-0.1(15.8)
FACT-G	3.4(74.0)*	-0.9(76.3)	3.0(75.9)*	-2.0(76.9)

Multiple linear regression adjusted for the potential influence of gender, age, BMI, education level, marital status, household income, time after diagnosis, treatment, comorbid chronic diseases and the number of comorbid chronic diseases.

Abbreviations: PA, physical activity; EORTC, QLQ-C30 the European Organization for Research and Treatment Quality of Life Questionnaires; PF, Physical Functioning; RF, Role Functioning; EF, Emotional Functioning; CF, Cognitive Functioning; SF, Social Functioning; QL, Global Health; FA, Fatigue; NV, Nausea and Vomiting; PA, Pain; DY, Dyspnea; SL, Insomnia; AP, Appetite Loss; CO, Constipation; DI, Diarrhea; FI, Financial Difficulties; FACT-G, Functional Assessment of Cancer Therapy—General Questionnaire; PWB, Physical Well-being; SWB, Social/family Well-being; EWB, Emotional Well-being; FWB, Functional Well-being.

^a^ The differences in mean QOL score between cancer survivors with a higher PA frequency and lower PA frequency.

^b^ Mean score of QOL measures among cancer survivors with lower PA frequency.

*p<0.05,

**p<0.01,

***p<0.001.

Lung cancer survivors with higher PA frequency generally reported lower scores on the symptom scales of the EORTC QLQ-C30, indicating a lower level of problems or symptoms. However, the association between QOL and PA frequency was not statistically significant among gynecological, colorectal or liver cancer survivors. Moreover, poorer QOL scores for several scales were observed among survivors of these three cancer types who had higher PA frequency.

The association between PA frequency and QOL remained undefined among lung, gynecological, cervical and liver cancer survivors.

## Discussion

Previous evidence has shown that demographic characteristics such as age, gender, education level, and marital status are important factors associated with QOL among cancer survivors [24–26]. In addition, CCD has been recently demonstrated to negatively influence QOL of patients diagnosed with different types of cancer, such as lung [19], gynecological [27], and breast cancers [28]. Treatment may also impact the QOL among cancer survivors [29, 30]. According to the results of this study, surgery improved the overall QOL among lung and liver cancer survivors. Because the main goal of this research was to investigate QOL and its association with PA, multiple linear regression models were applied to control the potential confounding factors.

Based on the results shown in Table 2, lung cancer survivors performed worse than other cancer survivors in physical functioning and dyspnea in this study. Colorectal cancer survivors claimed the most severe constipation and diarrhea and a high level of appetite loss. Liver cancer survivors reported the worst social functioning scores, global health status, and emotional well-being, as well as the highest level of appetite loss. Of course, all of the results were comparisons only between the four types of cancer survivors evaluated in this study and require further support. As limited types of cancer survivors were recruited, the extension of all these conclusions requires further study to compare the QOL of cancer survivors included with that of survivors from other cancer types not evaluated in this study. Better understanding of the QOL differences among survivors of different cancer types can help clinicians and public health service providers deliver more targeted health promotion programs. However, few studies have compared the differences of QOL between survivors of different cancer types.

Many investigations have been conducted to assess the relationship between PA and QOL measures among cancer survivors. For instance, PA may be an effective intervention to improve cognitive functioning for both cognitively healthy and impaired populations [31, 32]. According to the results of our study, survivors with PA reported significant better cognitive functioning among lung, gynecological and colorectal cancer survivors. However, such association was not observed among liver cancer survivors. This discrepancy regarding the relationship between PA and cognitive functioning was just an example of the varying degrees of that between PA and QOL measures among patients with different types of cancers. Additionally, although survivors with PA reported significant improvements on QOL measures among lung, gynecological or colorectal cancer survivors, these associations were not statistically significant for liver cancer survivors.

Moreover, the relationship between PA frequency and QOL remained uncertain in this study. According to Table 4, gynecological cancer survivors with higher PA frequency reported poorer QOL in role functioning, social functioning, and global health status, as well as on many symptom scales, although most of these influences were not statistically significant. Similar results were observed among colorectal and liver cancer survivors, among whom higher PA frequency might be related to poorer QOL conditions for some measures. In addition, the associations between the increased frequency of PA and physical functioning or physical well-being among gynecological or liver cancer survivors were not observed. To better compare the relationship between time spent in PA and QOL among colorectal cancer survivors, Van Roekel [33] applied linear regression models to calculate the adjusted mean differences of QOL measures between the light physical activity (LPA) and moderate-to-vigorous physical activity (MVPA) quartiles. A higher quartile indicates a higher PA frequency. According to the results of that study, although both LPA and MVPA beneficially impacted QOL measures to some extent, there were significant improvements between the first and third quartile, instead of the fourth quartile, of LPA and MVPA. This implied a potential nonlinear association between PA frequency and QOL among colorectal cancer patients. However, in our study, survivors with higher PA frequency did not report better QOL for most of the measures among colorectal cancer survivors. There are several possible reasons that account for the divergence of our results. First, PA frequency in this study was roughly divided into two categories of “1–4 times/week” and “≥5 times/week”. To better assess the relationship between QOL and PA frequency, the categories of frequency should be increased. Additionally, differences could have been caused because the studies evaluated different study populations and our study only used self-reported QOL questionnaires.

In this study, we tentatively suggest that both QOL and its association with PA vary among survivors of different cancer types. This conclusion may be valuable to public health practitioners, who can improve the current health care management for cancer survivors of specific cancer types according to the results of this study. For instance, we cautiously advocate that a higher frequency of PA is not strongly suggested to gynecological, colorectal or liver cancer survivors.

We have to acknowledge that there are some limitations of this study. First, light physical activity was not taken into consideration, thus we cannot generalize our conclusions. Additionally, the frequency of PA was only divided into two categories, and therefore, we could not report more detailed data about the relationship between PA frequency and QOL.

The major strengthen of this study was its large sample size. We recruited 3392 subjects in total, which ensured the representation of many cancer types in the sample. Moreover, survivors of six cancer types were included in this research, making it feasible to compare the potential differences of the relationship between QOL and PA. Furthermore, this study can aid and inspire other investigators to assess these potential differences among patients diagnosed with other types of cancer.

Further studies are warranted to refine the comparison of the relationship between QOL and PA among survivors of other cancer types. We suggest that future studies should evaluate the effect of PA type and frequency on this relationship in greater detail.

The conception of this article was inspired by the contradiction between the results from our published article [19] and those of several other studies [20, 21]. In the previously published article, we found association between PA and QOL among lung cancer survivors. However, this conclusion might contradict the results reported for survivors of other cancer types, which motivated us to explore the relationship. Although the same lung cancer participant population was used in both of our studies, there were two main differences that distinguish them. First, CCDs were treated as potential confounding factors in this article, whereas they were critical factors to be investigated in the previous study. Second, the main goal of this study was to compare the association between QOL and PA among survivors of different cancers, while the previous article investigated the relationship between CCDs/PA and QOL among lung cancer survivors. The gynecological cancer (including cervical, ovarian and endometrial cancers) survivors in this article also shared the same participant population with another article [27]. The main goal of that article was to investigate the relationship between CCDs and QOL among gynecological cancer survivors, and PA was not taken into consideration, unlike in the current study. The results were presented in separate articles for three main reasons. First, the different objectives of these articles made it impossible to consolidate all the data into one cohesive article. Second, the main goal of this study was to compare QOL among survivors of different cancers, and therefore, we wanted to include as many types of cancer survivors as we could. Last, this article was inspired by our previous article on lung cancer survivors.

## Supporting Information

S1 File EORTC QLQ-C30 (version 3.0). (PDF)

S2 File FACT-G. (PDF)
