# Supplementary material for: Quality of Life and Its Association with Physical Activity among Different Types of Cancer Survivors
Source: PLoS One. 2016 Nov 3;11(11):e0164971. doi: 10.1371/journal.pone.0164971 (PMC5094752; doi:10.1371/journal.pone.0164971)
Supplement: S2 File — (PDF) [file pone.0164971.s002.pdf]

FACT-G (Version 4)

Below is a list of statements that other people with your illness have said are important. **Please circle or mark one number per line to indicate your response as it applies to the past 7 days.**

**PHYSICAL WELL-BEING**	Not at all	A little bit	Somewhat	Quite a bit	Very much
I have a lack of energy	0	1	2	3	4
I have nausea	0	1	2	3	4
Because of my physical condition, I have trouble meeting the needs of my family	0	1	2	3	4
I have pain	0	1	2	3	4
I am bothered by side effects of treatment	0	1	2	3	4
I feel ill	0	1	2	3	4
I am forced to spend time in bed	0	1	2	3	4

**SOCIAL/FAMILY WELL-BEING**	Not at all	A little bit	Somewhat	Quite a bit	Very much
I feel close to my friends	0	1	2	3	4
I get emotional support from my family	0	1	2	3	4
I get support from my friends	0	1	2	3	4
My family has accepted my illness	0	1	2	3	4
I am satisfied with family communication about my illness	0	1	2	3	4
I feel close to my partner (or the person who is my main support)	0	1	2	3	4
I am satisfied with my sex life	0	1	2	3	4

**EMOTIONAL WELL-BEING**	Not at all	A little bit	Somewhat	Quite a bit	Very much
I feel sad	0	1	2	3	4
I am satisfied with how I am coping with my illness	0	1	2	3	4
I am losing hope in the fight against my illness	0	1	2	3	4
I feel nervous	0	1	2	3	4
I worry about dying	0	1	2	3	4
I worry that my condition will get worse	0	1	2	3	4

**FUNCTIONAL WELL-BEING**	Not at all	A little bit	Somewhat	Quite a bit	Very much
I am able to work (include work at home)	0	1	2	3	4
My work (include work at home) is fulfilling	0	1	2	3	4
I am able to enjoy life	0	1	2	3	4
I have accepted my illness	0	1	2	3	4
I am sleeping well	0	1	2	3	4
I am enjoying the things I usually do for fun	0	1	2	3	4
I am content with the quality of my life right now	0	1	2	3	4
